# Biological Sex: A Potential Moderator of Physical Activity Efficacy on Brain Health

**DOI:** 10.3389/fnagi.2019.00329

**Published:** 2019-12-06

**Authors:** Cindy K. Barha, Chun-Liang Hsu, Lisanne ten Brinke, Teresa Liu-Ambrose

**Affiliations:** ^1^Department of Physical Therapy, University of British Columbia, Vancouver, BC, Canada; ^2^Djavad Mowafaghian Centre for Brain Health, University of British Columbia, Vancouver, BC, Canada; ^3^Centre for Hip Health and Mobility, Vancouver Coastal Health Research Institute, Vancouver, BC, Canada

**Keywords:** sex differences, exercise, physical activity, cognition, hippocampus, prefrontal cortex, aging, dementia

## Abstract

The number of older people worldwide living with cognitive impairment and neurodegenerative diseases is growing at an unprecedented rate. Despite accumulating evidence that engaging in physical activity is a promising primary behavioral strategy to delay or avert the deleterious effects of aging on brain health, a large degree of variation exists in study findings. Thus, before physical activity and exercise can be prescribed as “medicine” for promoting brain health, it is imperative to understand how different biological factors can attenuate or amplify the effects of physical activity on cognition at the individual level. In this review article, we briefly discuss the current state of the literature, examining the relationship between physical activity and brain health in older adults and we present the argument that biological sex is a potent moderator of this relationship. Additionally, we highlight some of the potential neurobiological mechanisms underlying this sex difference for this relatively new and rapidly expanding line of research.

## Introduction

The worldwide population is aging at an unprecedented rate, with the proportion of people aged over 60 years projected to increase from 12% in 2015 to 22% in 2050, resulting in over 2 billion people aged 60 and over (WHO, 2018). Normative aging is characterized by multifaceted changes in cognitive function and brain structure. In particular, the domains of memory and executive functions show declines in performance, which is further reflected in atrophy in the brain regions that subserve these cognitive domains, namely the hippocampus and the prefrontal cortex (PFC; Salthouse, 2011). In non-normative cognitive aging, decline is much more dramatic and is associated with increased dementia risk.

Dementia is one of the major causes of disability in older adults (ASC, 2010). Worldwide, over 47 million people have dementia and current projections estimate that this number will reach 74.7 million by the 2030 and 131.5 million by 2050 (Prince et al., 2015). Within this context, these demographic changes in the population will lead to increasing challenges for healthcare systems. Therefore, identifying strategies that bolster healthy cognitive aging is of upmost importance globally.

As there is no effective pharmaceutical treatment currently available for cognitive impairment and dementia, there is great interest in the utility of lifestyle approaches. Physical activity (PA) is a promising primary behavioral non-pharmacological strategy to delay or avert the deleterious effects of aging on brain health (Phillips, 2017; Petersen et al., 2018). However, before PA can be prescribed as “medicine” for promoting brain health in the diseased and non-diseased brain, it is imperative to understand how different biological factors can attenuate or amplify the effects of PA on cognition at the individual level.

One such endogenous factor that appears to moderate PA efficacy is biological sex (Barha et al., 2017a,c; Loprinzi and Frith, 2018). The need to assess potential sex differences in this context is further highlighted by the greater prevalence of mild cognitive impairment (MCI) in males (Petersen et al., 2010), but faster rates of progression from MCI to Alzheimer’s disease (AD) in females (Li et al., 2016; Podcasy and Epperson, 2016). Here, we briefly review the current evidence that PA and exercise promote cognitive health in older adults with and without cognitive impairment. We present supporting evidence that biological sex is an important moderator of the relationship between PA and cognition and highlight some of the potential underlying mechanisms for this relatively new and rapidly expanding line of inquiry. We have limited our review mainly to studies in humans. We refer readers to several excellent review articles of exercise studies conducted in rodents (Cotman et al., 2007; Voss et al., 2013; Patten et al., 2015; Triviño-Paredes et al., 2016; Barha et al., 2017b; Cooper et al., 2018).

## The Hippocampus and Prefrontal Cortex: Brain Structures Implicated in Brain Aging

The process of aging is accompanied by declines in memory and executive functions which is further reflected in atrophy in the brain regions that subserve these cognitive domains, namely the hippocampus and the PFC (Salthouse, 2011). Episodic memory, memory for events that occur in a specific place and time, shows age-associated decline (Rönnlund et al., 2005) and relies on the integrity of the hippocampus, a medial temporal lobe brain structure. Executive functions refer to the higher-level mental processes required to control, plan and coordinate other cognitive abilities and behaviors (Espy, 2004); typically executive functions are divided into three general abilities, inhibition, working memory, and cognitive flexibility (Diamond, 2013), and rely on the PFC. Gray matter volume declines with increasing age are most prominent in the PFC, with more moderate declines in the hippocampus (Terry and Katzman, 2001; Raz et al., 2004). Interestingly, 82.5% of the longitudinal age-associated reduction in the executive function of inhibition was explained by changes in structural and functional connectivity (Fjell et al., 2017). Sex differences in the rate of regional volume loss, including the frontal lobe and hippocampus, in older adults have been found, with females faring better (Armstrong et al., 2019), though not all studies find this (Raz et al., 2010; Persson et al., 2016).

## Physical Activity and Brain Health

PA, any bodily movement produced by skeletal muscles, can be categorized into occupational, sports, conditioning, household, or other types of activities requiring energy expenditure (Caspersen et al., 1985). Exercise is a subtype of PA that must be planned, structured and be repetitive with the goal of improving or maintaining physical fitness (Caspersen et al., 1985). Generally, there are two main types of exercise: (1) aerobic training (AT; e.g., walking, running) which aims to improve cardiovascular fitness; and (2) resistance training (RT; e.g., weightlifting) which aims to improve muscle mass and strength. The vast majority of the literature has focused on the effects of AT on cognitive health in older adults, although in more recent years the efficacy of RT has been shown (for examples see Cassilhas et al., 2007; Liu-Ambrose et al., 2010, 2012; Nagamatsu et al., 2012; Best et al., 2015; Bolandzadeh et al., 2015). Evidence from epidemiological and randomized controlled trials (RCTs) supports the notion that engaging in PA and exercise are promising strategies for dementia prevention and disease modification. In a meta-analysis, Northey et al. (2018) found that to maximize the effectiveness to improve cognition in older adults, exercise bouts should be 45–60 min in duration and at least done at a moderate intensity.

Prospective, epidemiological studies historically observe the association between the amount of PA engaged in and changes in cognitive performance or dementia risk. Overall, the evidence from these studies supports the relationship between higher levels of PA and better cognitive outcomes, reduced dementia risk, and longevity. A meta-analysis of 21 cohort studies found that in older, community-dwelling individuals, higher levels of PA were associated with a 35% reduction in the risk for cognitive decline compared to lower levels of PA (Blondell et al., 2014). These findings built upon an earlier meta-analysis of 15 prospective studies among participants without dementia that found higher levels of PA were associated with a 38% lower risk of cognitive decline, and low to moderate levels of PA were associated with a 35% reduction (Sofi et al., 2011). Higher levels of PA are also associated with a reduction in risk for dementia (~14%) as seen in a meta-analysis of 26 cohort studies (Blondell et al., 2014). The protective effects of high levels of PA may be more pronounced for those with AD compared to all-cause dementia and vascular dementia (Guure et al., 2017). Importantly, using data from the Netherlands Cohort Study, a higher total daily non-occupational PA was associated with greater odds of surviving to 90 years of age in men, and in women engaging in 60 min/day of non-occupational PA was associated with the greatest odds of surviving (Brandts and van den Brandt, 2019). Additionally, greater functional fitness is associated with better general cognition; however, the specific aspects of functional fitness that correlate with cognition differ between males and females (Guo et al., 2018).

Some epidemiological studies have also employed neuroimaging techniques to better understand the neural correlates underlying the relationship between PA and cognitive function in older age. For example, in adults 65 years and older at baseline, greater amounts of PA quantified as the number of blocks walked in 1 week at baseline, predicted greater gray matter volumes of the prefrontal, occipital, entorhinal, and hippocampal regions 9 years later, and this greater volume was related to a two-fold reduction in the risk for cognitive impairment at year 13 (Erickson et al., 2010). Further, engaging in PA in midlife was associated with larger total brain volume and gray matter volume of the frontal cortex 21 years later (Rovio et al., 2010). Importantly, better maintenance of PA levels over 10 years in older age is associated with less reductions in hippocampal volume, smaller increases in global gray matter mean diffusivity and white matter axial diffusivity, and maintenance of global cognitive function, independent of baseline levels of PA, demographics, and APOE4 status (Best et al., 2017). In individuals at risk for AD who experience greater rates of brain atrophy, engaging in PA levels that meet or exceed the current PA recommendations is associated with greater volumes of the inferior and anterior temporal lobes compared to those not meeting the PA recommendations (Dougherty et al., 2016). Thus, the association between how much PA is engaged in, and maintenance of cognition in older age appears related to sparing of gray matter volume of brain regions susceptible to age-related atrophy, including the frontal and prefrontal lobes and the hippocampus (Gordon et al., 2008).

Although prospective cohort studies typically include large samples of individuals, causality cannot be established and the potential for unmeasured confounding variables is often present. Thus, stronger evidence for the importance of PA and exercise in brain health has come from RCTs. The vast majority of RCTs of exercise have focused on AT, which we will focus on in this review. However it is worth noting that RT has been shown to significantly improve cognition and brain function (for a recent review see Landrigan et al., 2019). Several meta-analyses of RCTs in older adults suggest that cognitive processes that are highly susceptible to age-related declines are amendable to AT benefits (Heyn et al., 2004; Etnier et al., 2006; Barha et al., 2017a; Northey et al., 2018), particularly the domain of executive functions (Colcombe and Kramer, 2003), an umbrella term for a suite of higher order cognitive processes required for goal-directed behavior. A seminal study in the field of exercise neuroscience conducted by Kramer et al. (1999) found that older adults randomized to a 6 month AT intervention (i.e., brisk walking) showed significant improvements in executive functions compared to participants randomized to a stretching and toning control group. In a follow-up study, the AT group showed increased gray and white matter volumes in the temporal and prefrontal regions of the brain compared to the control group (Colcombe et al., 2006). In addition to improvements in executive functions, a 12-month RCT found that AT increased hippocampal volume by 2%, coinciding with improvements in spatial memory performance compared to the stretching and toning control group that showed a 1.4% decline in volume (Erickson et al., 2011), which is comparable to the 1–2% annual hippocampal shrinkage typically seen in older adults (Raz et al., 2005). More recently, several meta-analyses of RCTs have provided further support for the protective effects of AT on cognition in older adults (Colcombe and Kramer, 2003; Heyn et al., 2004; Etnier et al., 2006; Barha et al., 2017a; Northey et al., 2018). Notably, AT benefits for cognition are seen in cognitively healthy older adults as well as across different clinical populations, including MCI, vascular dementia, and AD (Lautenschlager et al., 2008; Baker et al., 2010; Erickson et al., 2011; Nagamatsu et al., 2013; Liu-Ambrose et al., 2016; Morris et al., 2017). RCTs with neuroimaging outcomes indicate that AT interventions also lead to enhanced functional brain plasticity as indexed by changes in brain structure, activation, and connectivity variables (Voss et al., 2010; Erickson et al., 2011; Nishiguchi et al., 2015; ten Brinke et al., 2015; Gajewski and Falkenstein, 2016; Hsu et al., 2017, 2018). Specifically for gray matter changes, RCTs of AT consistently show increases in the volume of the hippocampus (Colcombe et al., 2006; Erickson et al., 2011; Niemann et al., 2014; Maass et al., 2015; Kleemeyer et al., 2016; Rosano et al., 2017), which subserves memory, and the PFC (Colcombe et al., 2006; Erickson et al., 2010; Ruscheweyh et al., 2011; Tamura et al., 2015; Jonasson et al., 2017), which subserves executive functions.

Despite the large body of evidence supporting the therapeutic potential of AT, variation exists in the ability of exercise to prevent and alleviate declines in cognition and brain health. Some meta-analyses of RCTs do not conclude that AT exerts a significant positive effect on cognition in older adults (Smith et al., 2010; Gates et al., 2013; Kelly et al., 2014; Öhman et al., 2014; Young et al., 2015). Notably, one of the largest RCTs of exercise conducted to date, did not find a significant effect of 24-months of exercise on cognitive performance in 1,635 older adults compared to a health education program (Sink et al., 2015). More recently, Lamb et al. (2018) found that a 4 month program consisting of supervised moderate to high intensity AT and RT in combination with unsupervised home-based exercises was associated with slight declines in global cognition several months after the supervised training ended in older adults with moderate dementia. Thus, to better understand the variation seen in study outcomes, it is imperative to identify biological moderators that either attenuate or amplify the effects of exercise on brain health. This knowledge will allow for more efficient and targeted deployment of current PA interventions and potentially spur development of alternative strategies. We argue here that biological sex is a key moderator of aerobic exercise efficacy, such that females show greater cognitive benefits than males.

## Sex Differences, Physical Activity and Brain Health

Sex differences exist in the magnitude of beneficial effects seen in cognitive function from engaging in aerobic exercise (see Table 1). Meta-analytical evidence from Colcombe and Kramer (2003) first suggested that older females showed greater cognitive gains from AT than older males. Our recent meta-analysis of RCTs conducted with cognitively healthy older adults confirmed this, as AT was associated with larger effect sizes in studies that included a higher percentage of female participants compared to studies with a lower percentage of female participants for the cognitive domain of executive functions (Barha et al., 2017a). Sex differences were not seen for other domains, including episodic memory and visuospatial function. Interestingly, AT enhanced word fluency to a greater extent in studies with a lower percentage of female participants. Thus, for tasks associated with verbal learning and memory, AT may be more advantageous for males than females. Moreover, our meta-analysis of studies in middle-aged and aged rodents found a female-advantage in gains for hippocampus-dependent spatial learning and memory in studies that utilized forced AT paradigms (Barha et al., 2017b). Unfortunately, we could not examine potential sex differences in AT efficacy for executive functions as there was a lack of such studies conducted with rodents. Regardless, altogether, the meta-analytic evidence available thus far indirectly supports the notion that biological sex is an important moderator of the relationship between AT and cognition in older age.

**Table 1:** Evidence for sex differences in the effect of physical activity on cognition and the brain in older humans.

Study	Population	Study type and Physical activity	Results and conclusions
Baker et al. (2010)	- 15 older females with MCI - 14 older males with MCI	RCT - 6 months of high intensity AT - Stretching control	In females, AT improved performance in 4/5 tests of executive function (Symbol-Digit, Verbal Fluency, Stroop, Trails B). In males, AT only improved performance in 1/5 tests (Trails B).
Barha et al. (2017d)	- 29 older females with SIVCI - 29 older males with SIVCI	RCT - 6 months of moderate intensity AT - Usual care plus education control	In females, AT improved executive functioning on the Trail Making Test. In males, AT did not improve executive functioning.
Barha et al. (2019)	- 1,497 older females cognitively healthy (177 females with MRI) - 1,376 older males cognitively healthy (126 males with MRI)	Epidemiological cohort study - Self-reported time spent walking (mins/week) each year for 10 years	In females, maintaining PA over 10 years predicted less declines in executive functioning on the Digit Symbol Substitution Test and was associated with greater volume of the left dorsolateral prefrontal cortex. In males, maintaining PA did not predict executive functioning.
van Uffelen et al. (2008)	- 67 older females with MCI - 85 older males with MCI	RCT - 12 months of moderate intensity AT - Low intensity, non-aerobic balance and flexibility exercise control	In females, increased adherence to the AT program was associated with improved executive functioning on the Stroop test and on an auditory verbal memory test. In males, attendance in at least 75% of AT classes was required before improvements in only auditory verbal memory were seen.
Varma et al. (2015)	- 64 older females cognitively healthy - 28 older males cognitively healthy	Sub study of an RCT - Daily walking activity measuring using a step activity monitor worn on the dominant ankle for 3–7 days	In females, greater amount, duration, and frequency of total daily walking were all associated with larger hippocampal volume. In males, associations were not significant.

To date, a small number of human studies have attempted to directly compare males and females to determine whether AT effects on cognition are sex-dependent. In a study sample of 29 older adults (15 women) with MCI, Baker et al. (2010) showed that 6-months of high intensity AT increased performance on four of five tests of cognition in females and only on one test in males compared to the controls. Further, in a 12-month study of moderate intensity AT, sex stratified analyses indicated that increased adherence to the AT program was associated with improved attention and memory in older females with MCI but only with memory in older males (van Uffelen et al., 2008). More recently, we provided further support for a female advantage in AT-induced gains in executive functions with a secondary analysis of a 6-month RCT in participants with mild subcortical ischemic vascular cognitive impairment (Barha et al., 2017d). The progressive, moderate intensity AT program improved the executive function of set-shifting by 36% in females compared to the controls, whereas in males AT reduced performance by 31% compared to the controls. Interestingly, the beneficial effect of AT was retained 6 months after trial completion. Epidemiological evidence for this sex difference in AT efficacy was recently provided in our study that examined whether longitudinal changes in PA over 10 years predicted changes in global cognition, executive functions and processing speed differently in males and females using data from the Health, Aging, and Body Composition Study (HABC; Barha et al., 2019). Participants were 2,873 community-dwelling older adults aged 70–79 years at year 1 and PA was assessed annually from years 1–10 through self-reported time spent walking. Independent of demographics and disease-related variables, initial time spent walking and maintenance of PA over the 10 years predicted less declines in executive functions and processing speed as assessed by the Digit Symbol Substitution Test (DSST) in females but not males. Maintenance of PA over time predicted better global cognitive function on the Modified Mini-Mental Status Examination (3MS) in both males and females. Thus, accumulating evidence from intervention and epidemiological studies suggest that engaging in targeted exercise training of sufficient duration and intensity, as well as how PA levels change over time, protect different domains of cognition in females compared to males.

Investigations into the neural underpinnings of AT-induced enhancements have also shown potential sex differences. Varma et al. (2015) showed that greater amount, duration, and frequency of objectively measured daily walking over the span of 3–7 days were associated with larger hippocampal volume among older females but not males. In a follow-up study that explored hippocampal sub-regions, increased daily walking in females only was associated specifically with a larger subiculum which is part of the posterior hippocampus (Varma et al., 2016). We have recently expanded upon these findings showing that subjective assessment of time spent walking in year 1 of a 10 year study was associated with a larger left hippocampus in older males and a smaller hippocampus in older females (Barha et al., 2019). This discrepancy in the direction of the relationship between PA and hippocampal volume in females between studies may be related to differences in how walking behaviors were measured (i.e., objective vs. subjective) and the duration of measurement (i.e., 7 days vs. 10 years). Additionally, it may be the case that connectivity of the hippocampus to other brain regions may be a better indicator of functional performance than mere hippocampal volume (Burdette et al., 2010). Indeed, there is some evidence in the literature to support this as an AT intervention-induced increase in hippocampal volume in older females was associated with lower performance on an episodic memory task (ten Brinke et al., 2015). Thus, in addition to volumetric imaging, future studies should use multimodal techniques to delve further into this intriguing sex difference to focus on changes in functional connectivity of the hippocampus and its relationship to cognition and exercise.

In addition to the hippocampus, we have recently shown that greater maintenance of PA over 10 years is associated with a greater volume of the left dorsolateral PFC (DL-PFC) among older females, but not males (Barha et al., 2019). The DL-PFC subserves executive functions (Wagner et al., 2001), one of the domains in which PA improves performance in females (Baker et al., 2010; Barha et al., 2017d), and shows extensive volume loss in older age (Jernigan et al., 2001). Previous work also shows that AT is associated with a larger volume of this brain region (Erickson et al., 2010; Best et al., 2017) and the volume mediates the relationship between cardiovascular fitness level and performance on executive functioning tasks (Weinstein et al., 2012), though, vitally, sex differences were not assessed in these studies.

Notwithstanding the lack of understanding in the effects of PA on sex differences associated with the functional architecture of the brain, evidence suggests the presence of sex differences in the patterns of neural network coupling. For instance, compared to men, women seemed to exhibit stronger connectivity between amygdala and middle temporal gyrus, inferior frontal gyrus, postcentral gyrus and hippocampus (Kogler et al., 2016). Moreover, one cross-sectional study investigated whether difference in sex and hormonal fluctuations across menstrual cycle would impact functional connectivity patterns of the frontoparietal network (Hjelmervik et al., 2014). From examining 16 healthy younger women and 15 younger men, the study showed women typically displayed higher frontoparietal connectivity compared to men, irrespective of the influences exerted by fluctuating sex hormones through various phases in the menstrual cycle. Aligning with this finding, the UK Biobank study reported that across 2,750 female and 2,466 male participants between the age of 44–77 years, a significantly stronger connectivity in the default mode network was observed in females compared to males (Ritchie et al., 2018), for which the same findings were reported in a separate, multi-site community-based study conducted previously with 1,093 participants between 18–60 years old (Biswal et al., 2010). In 559 cognitively healthy adults 70 years and older, Jamadar et al. (2019) confirmed the finding of greater connectivity within the default mode network in females and further showed greater connectivity within the salience network in males, two important resting-state networks that consistently show age-associated connectivity declines (Geerligs et al., 2015). Further, examining time-varying properties of connectivity patterns, de Lacy et al. (2019) demonstrated that dynamic connectivity of neural networks is significantly different between males and females, which complements observations made by classic methods of quantifying functional connectivity. These results, in conjunction, offer insights into sex differences in the functional organization of the brain such that women may be more proficient in social, self-referential and memory processes, in line with conclusions derived from brain structural connectomics (Ingalhalikar et al., 2014).

## Potential Mechanisms Underlying the Sex Difference

The mechanisms underlying the sex difference in the relationship between PA and cognitive and brain health have only recently been speculated upon in the literature (Barha et al., 2017c; Barha and Liu-Ambrose, 2018; Loprinzi and Frith, 2018). A large body of evidence, stemming mainly from animal studies, highlights the importance of neurotrophic factors, in particular brain derived neurotrophic factor (BDNF), in the AT-induced enhancements in cognition, brain function and neuroplasticity (Cotman et al., 2007). BDNF supports neuroplasticity, synaptic plasticity and the cellular mechanisms involved in learning and memory (Leal et al., 2015) and has been the focus of several AT studies. Several rodent studies indicate that central BDNF levels, particularly within the hippocampus, mediate the beneficial effects of AT on the brain (Voss et al., 2011, 2013; Triviño-Paredes et al., 2016). Additionally, an acute bout of running increases BDNF within the PFC of male mice (Baranowski et al., 2017). In contrast, evidence linking AT and increases in circulating BDNF levels is less clear in humans, although the majority of studies do support the role of BDNF in mediating AT efficacy (Knaepen et al., 2010; Szuhany et al., 2015). Several potential reasons for this lack of consistency in the literature have been proposed, including: (i) length of time between AT and BDNF assessment; (ii) intensity of AT; (iii) duration of AT; (iv) BDNF Val66Met genotype; and (v) sex of participants (Coelho et al., 2013; Szuhany et al., 2015; Maass et al., 2016; Barha et al., 2017c). Sex differences do exist in some of the functions attributed to BDNF (Phillips et al., 2014; Leal et al., 2015; Atwi et al., 2016; Chan and Ye, 2017; Scharfman and MacLusky, 2017). Indeed, a meta-analysis of 29 AT studies of different exercise paradigms (AT and RT, different durations and lengths) in humans of any age showed that sex was a significant moderator such that studies with more males showed greater BDNF changes from exercise (Szuhany et al., 2015). In contrast to these findings, a meta-analysis of studies of AT in older rodents showed that BDNF gains were greater in studies utilizing female rodents compared to male rodents (Barha et al., 2017b). Further, we recently showed in older humans with SIVCI, that 6-months of moderate intensity AT increased circulating BDNF levels in females but not males (Barha et al., 2017d). Altogether, these findings highlight the need for further studies of biological sex differences in the contributions of neurotrophic factors to AT benefits for brain health.

In addition to neurotrophic factors, the sex difference in the PA-induced cognitive and brain region volume response, may be related to several other factors, including alterations in neuroplastic processes, hormones, neurotransmitter systems, and physiological adaptations to exercise. For example, AT induces changes in neurogenesis, synaptogenesis, and angiogenesis in key brain regions involved in learning and memory (Voss et al., 2013; Prakash et al., 2015; Duzel et al., 2016; Triviño-Paredes et al., 2016). Many of these neuroplastic processes show sex differences (Barha and Liu-Ambrose, 2018); thus, it is possible that AT effects on these outcomes may be sex-dependent.

There have been relatively few studies that examine sex steroid hormone responses to long term PA within the context of aging. In older sedentary men, a 6-week intervention of moderate intensity aerobic exercise alone (Hayes et al., 2015) and when followed by 6-weeks of high intensity interval training (Hayes et al., 2017) was associated with increased circulating levels of total testosterone, although not all studies have found this association (for a review of the literature, see Hayes and Elliott, 2019). In older healthy women, a meta-analysis of 18 RCTs found that PA, particularly of high intensity, was associated with a small but statistically significant decrease in circulating total estradiol, as well as reductions in free testosterone (Ennour-Idrissi et al., 2015). In contrast to circulating levels of sex hormones, local, tissue-specific synthesis of testosterone and estradiol may be of more importance for brain health. For example, an acute bout of running in young rodents led to increased levels of testosterone in skeletal muscles in both males and females, whereas levels of estradiol only increased in males (Aizawa et al., 2008). On the other hand, using a rodent model of surgical menopause, Shi et al. (2019) recently found that longer-term running led to increased levels of estradiol in skeletal muscle in females. Studies looking at the effects of PA on local synthesis of sex steroids within the brain are scarce. In young male rodents, low intensity running led to increased *de novo* synthesis of dihydrotestosterone, a non-aromatizable androgen, in the hippocampus (Okamoto et al., 2012). Significantly, in the same study, depletion of circulating levels of androgens *via* castration did not abolish the beneficial effect of running on the hippocampus, supporting the importance of locally synthesized sex steroids in PA-effects in brain function. In addition to sex steroid hormones, other hormones may be involved in the sex difference in PA effects on the brain. For example, recently Yüksel et al. (2019) showed that 6 weeks of voluntary running increased oxytocin levels in the brain and periphery in female mice, which correlated with increased empathy-like behavior. In male mice, oxytocin was only increased in the brain, and levels did not correlate with empathy-like behavior. Further, PA may be exerting its influence on the brain through alterations in the hypothalamic-pituitary-adrenal axis (for a review, see Chen et al., 2017), and there are well established sex differences within this axis (for a recent review, see Rincón-Cortés et al., 2019).

PA also influences different neurotransmitter systems (Vecchio et al., 2018), including the dopaminergic system. PA in the form of aerobic exercise has been shown to increase dopamine levels and dopamine transmission within the brain of rodents and humans, including the hippocampus and PFC (Fisher et al., 2013; Robertson et al., 2016; Ko et al., 2019). Further support for the link between PA and dopamine comes from studying common polymorphisms within dopamine-related genes that alter dopamine signaling in humans. For example, carriers of the catechol-O-methyltransferase (COMT) Val158Met polymorphism, which is associated with dysregulated levels of dopamine in the brain, showed less cognitive improvements in response to 17 weeks of exercise (Stroth et al., 2010). Importantly, there are known sex differences in the effect of the COMT Val158Met polymorphism on the brain (Gurvich and Rossell, 2015) as well as in dopaminergic neurotransmission (Riccardi et al., 2011).

Sex differences exist within the respiratory, musculoskeletal and cardiovascular systems, leading to sex differences in the physiological responses of these systems to PA (Deschenes and Kraemer, 2002; Sheel et al., 2004; Green et al., 2016), with females typically at a disadvantage (Sheel et al., 2004; Harms and Rosenkranz, 2008). Potentially compounding this is the fact that older females engage in less PA and are more sedentary than older males (Kaplan et al., 2001; Lee, 2005), which has greater consequences for processing speed in females (Fagot et al., 2019). Therefore, increasing engagement in PA may lead to more appreciable effects on cognition and brain health in older females than older males. For a more comprehensive review of potential sex differences in mechanisms underlying AT effects on the brain, please see Barha and Liu-Ambrose (2018).

## Concluding Remarks

Engaging in PA is a relatively simple, highly implementable, and cost-effective lifestyle intervention with great promise in affording neuroprotection against cognitive decline in both normative and non-normative aging. Despite many studies supporting the utility of PA in preserving brain health in older age, a large degree of variation in PA efficacy exists (Smith et al., 2010; Gates et al., 2013; Kelly et al., 2014; Öhman et al., 2014; Young et al., 2015). Garnering a greater understanding of the sources of this variation will be a fundamental step towards the ultimate goal of prescribing PA as “medicine” for brain health. In this review article, we argued that biological sex is a potent moderator of the effectiveness of PA for both cognitive and neural outcomes. Future studies that aim to understand the mechanisms underlying PAs effects on the brain should take these profound sex differences into consideration.

## Author Contributions

CB wrote the first draft of the manuscript. C-LH, LB, and TL-A wrote sections of the manuscript. All authors read and approved the submitted version.

## Conflict of Interest

The authors declare that the research was conducted in the absence of any commercial or financial relationships that could be construed as a potential conflict of interest.

## References

[B1] Aizawa K. Iemitsu M. Otsuki T. Maeda S. Miyauchi T. Mesaki N. (2008). Sex differences in steroidogenesis in skeletal muscle following a single bout of exercise in rats. J. Appl. Physiol. 104, 67–74. 10.1152/japplphysiol.00558.2007 17975125

[B2] Armstrong N. M. An Y. Beason-Held L. Doshi J. Erus G. Ferrucci L.. (2019). Sex differences in brain aging and predictors of neurodegeneration in cognitively healthy older adults. Neurobiol. Aging 81, 146–156. 10.1016/j.neurobiolaging.2019.05.020 31280118 PMC9310670

[B3] ASC (2010). Rising Tide: The Impact of Dementia on Canadian Society. Toronto, ON: Alzheimer Society of Canada.

[B4] Atwi S. McMahon D. Scharfman H. MacLusky N. J. (2016). Androgen modulation of hippocampal structure and function. Neuroscientist 22, 46–60. 10.1177/1073858414558065 25416742 PMC5002217

[B5] Baker L. D. Frank L. L. Foster-Schubert K. Green P. S. Wilkinson C. W. Mctiernan A.. (2010). Effects of aerobic exercise on mild cognitive impairment: a controlled trial. Arch. Neurol. 67, 71–79. 10.1001/archneurol.2009.307 20065132 PMC3056436

[B6] Baranowski B. P. Peppler W. T. MacPherson E. K. (2017). Acute exercise rescues cortex BDNF signaling in high fat fed male mice. FASEB J. 31:lb707.

[B8] Barha C. K. Best J. R. Rosano C. Yaffe K. Catov J. M. Liu-Ambrose T.. (2019). Sex-specific relationship between long-term maintenance of physical activity and cognition in the health ABC study: potential role of hippocampal and dorsolateral prefrontal cortex volume. J. Gerontol. A Biol. Sci. Med. Sci. [Epub ahead of print]. 10.1093/gerona/glz093 30958523 PMC7931854

[B9] Barha C. K. Davis J. C. Falck R. S. Nagamatsu L. S. Liu-Ambrose T. (2017a). Sex differences in exercise efficacy to improve cognition: a systematic review and meta-analysis of randomized controlled trials in older humans. Front. Neuroendocrinol. 46, 71–85. 10.1016/j.yfrne.2017.04.002 28442274

[B10] Barha C. K. Falck R. S. Davis J. C. Nagamatsu L. S. Liu-Ambrose T. (2017b). Sex differences in aerobic exercise efficacy to improve cognition: a systematic review and meta-analysis of studies in older rodents. Front. Neuroendocrinol. 46, 86–105. 10.1016/j.yfrne.2017.06.001 28614695

[B11] Barha C. K. Galea L. A. Nagamatsu L. S. Erickson K. I. Liu-Ambrose T. (2017c). Personalising exercise recommendations for brain health: considerations and future directions. Br. J. Sports Med. 51, 636–639. 10.1136/bjsports-2016-096710 27856411

[B12] Barha C. K. Hsiung G. R. Best J. R. Davis J. C. Eng J. J. Jacova C.. (2017d). Sex difference in aerobic exercise efficacy to improve cognition in older adults with vascular cognitive impairment: secondary analysis of a randomized controlled trial. J. Alzheimers Dis. 60, 1397–1410. 10.3233/JAD-170221 29036816

[B7] Barha C. K. Liu-Ambrose T. (2018). Exercise and the aging brain: considerations for sex differences. Brain Plast. 4, 53–63. 10.3233/BPL-180067 30564546 PMC6296261

[B13] Best J. R. Chiu B. K. Liang Hsu C. Nagamatsu L. S. Liu-Ambrose T. (2015). Long-term effects of resistance exercise training on cognition and brain volume in older women: results from a randomized controlled trial. J. Int. Neuropsychol. Soc. 21, 745–756. 10.1017/s1355617715000673 26581787

[B14] Best J. R. Rosano C. Aizenstein H. J. Tian Q. Boudreau R. M. Ayonayon H. N.. (2017). Long-term changes in time spent walking and subsequent cognitive and structural brain changes in older adults. Neurobiol. Aging 57, 153–161. 10.1016/j.neurobiolaging.2017.05.023 28648916 PMC5564417

[B15] Biswal B. B. Mennes M. Zuo X. N. Gohel S. Kelly C. Smith S. M.. (2010). Toward discovery science of human brain function. Proc. Natl. Acad. Sci. U S A 107, 4734–4739. 10.1073/pnas.0911855107 20176931 PMC2842060

[B16] Blondell S. J. Hammersley-Mather R. Veerman J. L. (2014). Does physical activity prevent cognitive decline and dementia? a systematic review and meta-analysis of longitudinal studies. BMC Public Health 14:510. 10.1186/1471-2458-14-510 24885250 PMC4064273

[B17] Bolandzadeh N. Tam R. Handy T. C. Nagamatsu L. S. Hsu C. L. Davis J. C.. (2015). Resistance training and white matter lesion progression in older women: exploratory analysis of a 12-month randomized controlled trial. J. Am. Geriatr. Soc. 63, 2052–2060. 10.1111/jgs.13644 26456233

[B18] Brandts L. van den Brandt P. A. (2019). Body size, non-occupational physical activity and the chance of reaching longevity in men and women: findings from the Netherlands Cohort Study. J Epidemiol. Community Health 73, 239–249. 10.1136/jech-2018-211410 30665909

[B19] Burdette J. H. Laurienti P. J. Espeland M. A. Morgan A. Telesford Q. Vechlekar C. D.. (2010). Using network science to evaluate exercise-associated brain changes in older adults. Front. Aging Neurosci. 2:23. 10.3389/fnagi.2010.00023 20589103 PMC2893375

[B20] Caspersen C. J. Powell K. E. Christenson G. M. (1985). Physical activity, exercise, and physical fitness: definitions and distinctions for health-related research. Public Health Rep. 100, 126–131. 3920711 PMC1424733

[B21] Cassilhas R. C. Viana V. A. Grassmann V. Santos R. T. Santos R. F. Tufik S.. (2007). The impact of resistance exercise on the cognitive function of the elderly. Med. Sci. Sports Exerc. 39, 1401–1407. 10.1249/mss.0b013e318060111f 17762374

[B22] Chan C. B. Ye K. (2017). Sex differences in brain-derived neurotrophic factor signaling and functions. J. Neurosci. Res. 95, 328–335. 10.1002/jnr.23863 27870419 PMC5271179

[B23] Chen C. Nakagawa S. An Y. Ito K. Kitaichi Y. Kusumi I. (2017). The exercise-glucocorticoid paradox: How exercise is beneficial to cognition, mood, and the brain while increasing glucocorticoid levels. Front. Neuroendocrinol. 44, 83–102. 10.1016/j.yfrne.2016.12.001 27956050

[B24] Coelho F. G. Gobbi S. Andreatto C. A. Corazza D. I. Pedroso R. V. Santos-Galduroz R. F. (2013). Physical exercise modulates peripheral levels of brain-derived neurotrophic factor (BDNF): a systematic review of experimental studies in the elderly. Arch. Gerontol. Geriatr. 56, 10–15. 10.1016/j.archger.2012.06.003 22749404

[B26] Colcombe S. J. Erickson K. I. Scalf P. E. Kim J. S. Prakash R. Mcauley E.. (2006). Aerobic exercise training increases brain volume in aging humans. J. Gerontol. A Biol. Sci. Med. Sci. 61, 1166–1170. 10.1093/gerona/61.11.1166 17167157

[B25] Colcombe S. Kramer A. F. (2003). Fitness effects on the cognitive function of older adults: a meta-analytic study. Psychol. Sci. 14, 125–130. 10.1111/1467-9280.t01-1-01430 12661673

[B27] Cooper C. Moon H. Y. van Praag H. (2018). On the run for hippocampal plasticity. Cold Spring Harb. Perspect. Med. 8:a029736. 10.1101/cshperspect.a029736 28495803 PMC5880155

[B28] Cotman C. W. Berchtold N. C. Christie L. A. (2007). Exercise builds brain health: key roles of growth factor cascades and inflammation. Trends Neurosci. 30, 464–472. 10.1016/j.tins.2007.06.011 17765329

[B29] de Lacy N. McCauley E. Kutz J. N. Calhoun V. D. (2019). Sex-related differences in intrinsic brain dynamism and their neurocognitive correlates. Neuroimage 202:116116. 10.1016/j.neuroimage.2019.116116 31446126

[B30] Deschenes M. R. Kraemer W. J. (2002). Performance and physiologic adaptations to resistance training. Am. J. Phys. Med. Rehabil. 81, S3–S16. 10.1097/00002060-200211001-00003 12409807

[B31] Diamond A. (2013). Executive functions. Annu. Rev. Psychol. 64, 135–168. 10.1146/annurev-psych-113011-143750 23020641 PMC4084861

[B32] Dougherty R. J. Ellingson L. D. Schultz S. A. Boots E. A. Meyer J. D. Lindheimer J. B.. (2016). Meeting physical activity recommendations may be protective against temporal lobe atrophy in older adults at risk for Alzheimer’s disease. Alzheimers Dement. Amst. 4, 14–17. 10.1016/j.dadm.2016.03.005 27489874 PMC4950614

[B33] Duzel E. van Praag H. Sendtner M. (2016). Can physical exercise in old age improve memory and hippocampal function? Brain 139, 662–673. 10.1093/brain/awv407 26912638 PMC4766381

[B34] Ennour-Idrissi K. Maunsell E. Diorio C. (2015). Effect of physical activity on sex hormones in women: a systematic review and meta-analysis of randomized controlled trials. Breast Cancer Res. 17:139. 10.1186/s13058-015-0647-3 26541144 PMC4635995

[B35] Erickson K. I. Raji C. A. Lopez O. L. Becker J. T. Rosano C. Newman A. B.. (2010). Physical activity predicts gray matter volume in late adulthood: the Cardiovascular Health Study. Neurology 75, 1415–1422. 10.1212/WNL.0b013e3181f88359 20944075 PMC3039208

[B36] Erickson K. I. Voss M. W. Prakash R. S. Basak C. Szabo A. Chaddock L.. (2011). Exercise training increases size of hippocampus and improves memory. Proc. Natl. Acad. Sci. U S A 108, 3017–3022. 10.1073/pnas.1015950108 21282661 PMC3041121

[B37] Espy K. A. (2004). Using developmental, cognitive and neuroscience approaches to understand executive control in young children. Dev. Neuropsychol. 26, 379–384. 10.4324/9780203764428-1 15276900

[B38] Etnier J. L. Nowell P. M. Landers D. M. Sibley B. A. (2006). A meta-regression to examine the relationship between aerobic fitness and cognitive performance. Brain Res. Rev. 52, 119–130. 10.1016/j.brainresrev.2006.01.002 16490256

[B39] Fagot D. Chicherio C. Albinet C. T. Andre N. Audiffren M. (2019). The impact of physical activity and sex differences on intraindividual variability in inhibitory performance in older adults. Neuropsychol. Dev. Cogn. B Aging Neuropsychol. Cogn. 26, 1–23. 10.1080/13825585.2017.1372357 28868969

[B40] Fisher B. E. Li Q. Nacca A. Salem G. J. Song J. Yip J.. (2013). Treadmill exercise elevates striatal dopamine D2 receptor binding potential in patients with early Parkinson’s disease. Neuroreport 24, 509–514. 10.1097/wnr.0b013e328361dc13 23636255

[B41] Fjell A. M. Sneve M. H. Grydeland H. Storsve A. B. Walhovd K. B. (2017). The disconnected brain and executive function decline in aging. Cereb. Cortex 27, 2303–2317. 10.1093/cercor/bhw082 27073220

[B42] Gajewski P. D. Falkenstein M. (2016). Physical activity and neurocognitive functioning in aging - a condensed updated review. Eur. Rev. Aging Phys. Act. 13:1. 10.1186/s11556-016-0161-3 26865880 PMC4748322

[B43] Gates N. Fiatarone Singh M. A. Sachdev P. S. Valenzuela M. (2013). The effect of exercise training on cognitive function in older adults with mild cognitive impairment: a meta-analysis of randomized controlled trials. Am. J. Geriatr. Psychiatry 21, 1086–1097. 10.1016/j.jagp.2013.02.018 23831175

[B44] Geerligs L. Renken R. J. Saliasi E. Maurits N. M. Lorist M. M. (2015). A brain-wide study of age-related changes in functional connectivity. Cereb. Cortex 25, 1987–1999. 10.1093/cercor/bhu012 24532319

[B45] Gordon B. A. Rykhlevskaia E. I. Brumback C. R. Lee Y. Elavsky S. Konopack J. F.. (2008). Neuroanatomical correlates of aging, cardiopulmonary fitness level and education. Psychophysiology 45, 825–838. 10.1111/j.1469-8986.2008.00676.x 18627534 PMC5287394

[B46] Green D. J. Hopkins N. D. Jones H. Thijssen D. H. Eijsvogels T. M. Yeap B. B. (2016). Sex differences in vascular endothelial function and health in humans: impacts of exercise. Exp. Physiol. 101, 230–242. 10.1113/ep085367 26663420

[B47] Guo Y. Yang M. Yan Y. Wang L. Gong J. (2018). Sex differentials in relationships between functional fitness and cognitive performance in older adults: a canonical correlation analysis. Sci. Rep. 8:4146. 10.1038/s41598-018-22475-7 29515144 PMC5841399

[B48] Gurvich C. Rossell S. L. (2015). Dopamine and cognitive control: sex-by-genotype interactions influence the capacity to switch attention. Behav. Brain Res. 281, 96–101. 10.1016/j.bbr.2014.11.045 25510197

[B49] Guure C. B. Ibrahim N. A. Adam M. B. Said S. M. (2017). Impact of physical activity on cognitive decline, dementia and its subtypes: meta-analysis of prospective studies. Biomed. Res. Int. 2017:9016924. 10.1155/2017/9016924 28271072 PMC5320071

[B50] Harms C. A. Rosenkranz S. (2008). Sex differences in pulmonary function during exercise. Med. Sci. Sports Exerc. 40, 664–668. 10.1249/mss.0b013e3181621325 18317379

[B51] Hayes L. D. Elliott B. T. (2019). Short-term exercise training inconsistently influences basal testosterone in older men: a systematic review and meta-analysis. Front. Physiol. 9:1878. 10.3389/fphys.2018.01878 30692929 PMC6339914

[B52] Hayes L. D. Herbert P. Sculthorpe N. F. Grace F. M. (2017). Exercise training improves free testosterone in lifelong sedentary aging men. Endocr. Connect. 6, 306–310. 10.1530/ec-17-0082 28515052 PMC5510446

[B53] Hayes L. D. Sculthorpe N. Herbert P. Baker J. S. Spagna R. Grace F. M. (2015). Six weeks of conditioning exercise increases total, but not free testosterone in lifelong sedentary aging men. Aging Male 18, 195–200. 10.3109/13685538.2015.1046123 26030347

[B54] Heyn P. Abreu B. C. Ottenbacher K. J. (2004). The effects of exercise training on elderly persons with cognitive impairment and dementia: a meta-analysis. Arch. Phys. Med. Rehabil. 85, 1694–1704. 10.1016/j.apmr.2004.03.019 15468033

[B55] Hjelmervik H. Hausmann M. Osnes B. Westerhausen R. Specht K. (2014). Resting states are resting traits—an FMRI study of sex differences and menstrual cycle effects in resting state cognitive control networks. PLoS One 9:e103492. 10.1371/journal.pone.0103492 25057823 PMC4110030

[B56] Hsu C. L. Best J. R. Davis J. C. Nagamatsu L. S. Wang S. Boyd L. A.. (2018). Aerobic exercise promotes executive functions and impacts functional neural activity among older adults with vascular cognitive impairment. Br. J. Sports Med. 52, 184–191. 10.1136/bjsports-2016-096846 28432077

[B57] Hsu C. L. Best J. R. Wang S. Voss M. W. Hsiung R. G. Y. Munkacsy M.. (2017). The impact of aerobic exercise on fronto-parietal network connectivity and its relation to mobility: an exploratory analysis of a 6-month randomized controlled trial. Front. Hum. Neurosci. 11:449. 10.3389/fnhum.2017.00449 28900392 PMC5591871

[B58] Ingalhalikar M. Smith A. Parker D. Satterthwaite T. D. Elliott M. A. Ruparel K.. (2014). Sex differences in the structural connectome of the human brain. Proc. Natl. Acad. Sci. U S A 111, 823–828. 10.1073/pnas.1316909110 24297904 PMC3896179

[B59] Jamadar S. D. Sforazzini F. Raniga P. Ferris N. J. Paton B. Bailey M. J.. (2019). Sexual dimorphism of resting-state network connectivity in healthy ageing. J. Gerontol. B Psychol. Sci. Soc. Sci. 74, 1121–1131. 10.1093/geronb/gby004 29471348 PMC6748717

[B60] Jernigan T. L. Archibald S. L. Fennema-Notestine C. Gamst A. C. Stout J. C. Bonner J.. (2001). Effects of age on tissues and regions of the cerebrum and cerebellum. Neurobiol. Aging 22, 581–594. 10.1016/s0197-4580(01)00217-2 11445259

[B61] Jonasson L. S. Nyberg L. Kramer A. F. Lundquist A. Riklund K. Boraxbekk C. J. (2017). Aerobic exercise intervention, cognitive performance and brain structure: results from the physical influences on brain in aging (PHIBRA) study. Front. Aging Neurosci. 8:336. 10.3389/fnagi.2016.00336 28149277 PMC5241294

[B62] Kaplan M. S. Newsom J. T. Mcfarland B. H. Lu L. (2001). Demographic and psychosocial correlates of physical activity in late life. Am. J. Prev. Med. 21, 306–312. 10.1016/s0749-3797(01)00364-6 11701302

[B63] Kelly M. E. Loughrey D. Lawlor B. A. Robertson I. H. Walsh C. Brennan S. (2014). The impact of exercise on the cognitive functioning of healthy older adults: a systematic review and meta-analysis. Ageing Res. Rev. 16, 12–31. 10.1016/j.arr.2014.05.002 24862109

[B64] Kleemeyer M. M. Kuhn S. Prindle J. Bodammer N. C. Brechtel L. Garthe A.. (2016). Changes in fitness are associated with changes in hippocampal microstructure and hippocampal volume among older adults. Neuroimage 131, 155–161. 10.1016/j.neuroimage.2015.11.026 26584869

[B65] Knaepen K. Goekint M. Heyman E. M. Meeusen R. (2010). Neuroplasticity—exercise-induced response of peripheral brain-derived neurotrophic factor: a systematic review of experimental studies in human subjects. Sports Med. 40, 765–801. 10.2165/11534530-000000000-00000 20726622

[B66] Ko I. G. Kim C. J. Kim H. (2019). Treadmill exercise improves memory by up-regulating dopamine and down-regulating D2 dopamine receptor in traumatic brain injury rats. J. Exerc. Rehabil. 15, 504–511. 10.12965/jer.1938316.158 31523669 PMC6732546

[B67] Kogler L. Muller V. I. Seidel E. M. Boubela R. Kalcher K. Moser E.. (2016). Sex differences in the functional connectivity of the amygdalae in association with cortisol. Neuroimage 134, 410–423. 10.1016/j.neuroimage.2016.03.064 27039701 PMC6594554

[B68] Kramer A. F. Hahn S. Cohen N. J. Banich M. T. Mcauley E. Harrison C. R.. (1999). Ageing, fitness and neurocognitive function. Nature 400, 418–419. 10.1038/22682 10440369

[B69] Lamb S. E. Sheehan B. Atherton N. Nichols V. Collins H. Mistry D.. (2018). Dementia and Physical Activity (DAPA) trial of moderate to high intensity exercise training for people with dementia: randomised controlled trial. BMJ 361:k1675. 10.1136/bmj.k1675 29769247 PMC5953238

[B70] Landrigan J. F. Bell T. Crowe M. Clay O. J. Mirman D. (2019). Lifting cognition: a meta-analysis of effects of resistance exercise on cognition. Psychol. Res. [Epub ahead of print]. 10.1007/s00426-019-01145-x 30627769

[B71] Lautenschlager N. T. Cox K. L. Flicker L. Foster J. K. Van Bockxmeer F. M. Xiao J.. (2008). Effect of physical activity on cognitive function in older adults at risk for Alzheimer disease: a randomized trial. JAMA 300, 1027–1037. 10.1001/jama.300.9.1027 18768414

[B72] Leal G. Afonso P. M. Salazar I. L. Duarte C. B. (2015). Regulation of hippocampal synaptic plasticity by BDNF. Brain Res. 1621, 82–101. 10.1016/j.brainres.2014.10.019 25451089

[B73] Lee Y. S. (2005). Gender differences in physical activity and walking among older adults. J. Women Aging 17, 55–70. 10.1300/j074v17n01_05 15914419

[B74] Li J. Q. Tan L. Wang H. F. Tan M. S. Tan L. Xu W.. (2016). Risk factors for predicting progression from mild cognitive impairment to Alzheimer’s disease: a systematic review and meta-analysis of cohort studies. J. Neurol. Neurosurg. Psychiatry 87, 476–484. 10.1136/jnnp-2014-310095 26001840

[B75] Liu-Ambrose T. Best J. R. Davis J. C. Eng J. J. Lee P. E. Jacova C.. (2016). Aerobic exercise and vascular cognitive impairment: a randomized controlled trial. Neurology 87, 2082–2090. 10.1212/WNL.0000000000003332 27760869 PMC5109938

[B76] Liu-Ambrose T. Nagamatsu L. S. Graf P. Beattie B. L. Ashe M. C. Handy T. C. (2010). Resistance training and executive functions: a 12-month randomized controlled trial. Arch. Intern. Med. 170, 170–178. 10.1001/archinternmed.2009.494 20101012 PMC3448565

[B77] Liu-Ambrose T. Nagamatsu L. S. Voss M. W. Khan K. M. Handy T. C. (2012). Resistance training and functional plasticity of the aging brain: a 12-month randomized controlled trial. Neurobiol. Aging 33, 1690–1698. 10.1016/j.neurobiolaging.2011.05.010 21741129

[B78] Loprinzi P. D. Frith E. (2018). The role of sex in memory function: considerations and recommendations in the context of exercise. J. Clin. Med. 7:E132. 10.3390/jcm7060132 29857518 PMC6028920

[B79] Maass A. Duzel S. Brigadski T. Goerke M. Becke A. Sobieray U.. (2016). Relationships of peripheral IGF-1, VEGF and BDNF levels to exercise-related changes in memory, hippocampal perfusion and volumes in older adults. Neuroimage 131, 142–154. 10.1016/j.neuroimage.2015.10.084 26545456

[B80] Maass A. Duzel S. Goerke M. Becke A. Sobieray U. Neumann K.. (2015). Vascular hippocampal plasticity after aerobic exercise in older adults. Mol. Psychiatry 20, 585–593. 10.1038/mp.2014.114 25311366

[B81] Morris J. K. Vidoni E. D. Johnson D. K. Van Sciver A. Mahnken J. D. Honea R. A.. (2017). Aerobic exercise for Alzheimer’s disease: a randomized controlled pilot trial. PLoS One 12:e0170547. 10.1371/journal.pone.0170547 28187125 PMC5302785

[B82] Nagamatsu L. S. Chan A. Davis J. C. Beattie B. L. Graf P. Voss M. W.. (2013). Physical activity improves verbal and spatial memory in older adults with probable mild cognitive impairment: a 6-month randomized controlled trial. J. Aging Res. 2013:861893. 10.1155/2013/861893 23509628 PMC3595715

[B83] Nagamatsu L. S. Handy T. C. Hsu C. L. Voss M. Liu-Ambrose T. (2012). Resistance training promotes cognitive and functional brain plasticity in seniors with probable mild cognitive impairment. Arch. Intern. Med. 172, 666–668. 10.1001/archinternmed.2012.379 22529236 PMC3514552

[B84] Niemann C. Godde B. Voelcker-Rehage C. (2014). Not only cardiovascular, but also coordinative exercise increases hippocampal volume in older adults. Front. Aging Neurosci. 6:170. 10.3389/fnagi.2014.00170 25165446 PMC4131191

[B85] Nishiguchi S. Yamada M. Tanigawa T. Sekiyama K. Kawagoe T. Suzuki M.. (2015). A 12-week physical and cognitive exercise program can improve cognitive function and neural efficiency in community-dwelling older adults: a randomized controlled trial. J. Am. Geriatr. Soc. 63, 1355–1363. 10.1111/jgs.13481 26114906

[B86] Northey J. M. Cherbuin N. Pumpa K. L. Smee D. J. Rattray B. (2018). Exercise interventions for cognitive function in adults older than 50: a systematic review with meta-analysis. Br. J. Sports Med. 52, 154–160. 10.1136/bjsports-2016-096587 28438770

[B87] Öhman H. Savikko N. Strandberg T. E. Pitkälä K. H. (2014). Effect of physical exercise on cognitive performance in older adults with mild cognitive impairment or dementia: a systematic review. Dement. Geriatr. Cogn. Disord. 38, 347–365. 10.1159/000365388 25171577

[B88] Okamoto M. Hojo Y. Inoue K. Matsui T. Kawato S. Mcewen B. S.. (2012). Mild exercise increases dihydrotestosterone in hippocampus providing evidence for androgenic mediation of neurogenesis. Proc. Natl. Acad. Sci. U S A 109, 13100–13105. 10.1073/pnas.1210023109 22807478 PMC3420174

[B89] Patten A. R. Yau S. Y. Fontaine C. J. Meconi A. Wortman R. C. Christie B. R. (2015). The benefits of exercise on structural and functional plasticity in the rodent hippocampus of different disease models. Brain Plast. 1, 97–127. 10.3233/bpl-150016 29765836 PMC5928528

[B90] Persson N. Ghisletta P. Dahle C. L. Bender A. R. Yang Y. Yuan P.. (2016). Regional brain shrinkage and change in cognitive performance over two years: the bidirectional influences of the brain and cognitive reserve factors. Neuroimage 126, 15–26. 10.1016/j.neuroimage.2015.11.028 26584866 PMC4733615

[B91] Petersen R. C. Lopez O. Armstrong M. J. Getchius T. S. D. Ganguli M. Gloss D.. (2018). Practice guideline update summary: mild cognitive impairment: report of the guideline development, dissemination and implementation subcommittee of the American academy of neurology. Neurology 90, 126–135. 10.1212/wnl.0000000000006040 29282327 PMC5772157

[B92] Petersen R. C. Roberts R. O. Knopman D. S. Geda Y. E. Cha R. H. Pankratz V. S.. (2010). Prevalence of mild cognitive impairment is higher in men. The Mayo Clinic Study of Aging. Neurology 75, 889–897. 10.1212/WNL.0b013e3181f11d85 20820000 PMC2938972

[B93] Phillips C. (2017). Lifestyle modulators of neuroplasticity: how physical activity, mental engagement, and diet promote cognitive health during aging. Neural Plast. 2017:3589271. 10.1155/2017/3589271 28695017 PMC5485368

[B94] Phillips C. Baktir M. A. Srivatsan M. Salehi A. (2014). Neuroprotective effects of physical activity on the brain: a closer look at trophic factor signaling. Front. Cell. Neurosci. 8:170. 10.3389/fncel.2014.00170 24999318 PMC4064707

[B95] Podcasy J. L. Epperson C. N. (2016). Considering sex and gender in Alzheimer disease and other dementias. Dialogues Clin. Neurosci. 18, 437–446. 28179815 10.31887/DCNS.2016.18.4/cepperson PMC5286729

[B96] Prakash R. S. Voss M. W. Erickson K. I. Kramer A. F. (2015). Physical activity and cognitive vitality. Annu. Rev. Psychol. 66, 769–797. 10.1146/annurev-psych-010814-015249 25251492

[B97] Prince M. Wimo A. Guerchet M. Ali G. C. Wu Y. T. Prina M. (2015). World Alzheimer Report 2015. London: Alzheimer’s Disease International (ADI).

[B98] Raz N. Ghisletta P. Rodrigue K. M. Kennedy K. M. Lindenberger U. (2010). Trajectories of brain aging in middle-aged and older adults: regional and individual differences. Neuroimage 51, 501–511. 10.1016/j.neuroimage.2010.03.020 20298790 PMC2879584

[B99] Raz N. Lindenberger U. Rodrigue K. M. Kennedy K. M. Head D. Williamson A.. (2005). Regional brain changes in aging healthy adults: general trends, individual differences and modifiers. Cereb. Cortex 15, 1676–1689. 10.1093/cercor/bhi044 15703252

[B100] Raz N. Rodrigue K. M. Head D. Kennedy K. M. Acker J. D. (2004). Differential aging of the medial temporal lobe: a study of a five-year change. Neurology 62, 433–438. 10.1212/01.wnl.0000106466.09835.46 14872026

[B101] Riccardi P. Park S. Anderson S. Doop M. Ansari M. S. Schmidt D.. (2011). Sex differences in the relationship of regional dopamine release to affect and cognitive function in striatal and extrastriatal regions using positron emission tomography and [^18^F]fallypride. Synapse 65, 99–102. 10.1002/syn.20822 20506565 PMC2965297

[B102] Rincón-Cortés M. Herman J. P. Lupien S. Maguire J. Shansky R. M. (2019). Stress: influence of sex, reproductive status and gender. Neurobiol. Stress 10:100155. 10.1016/j.ynstr.2019.100155 30949564 PMC6430637

[B103] Ritchie S. J. Cox S. R. Shen X. Lombardo M. V. Reus L. M. Alloza C.. (2018). Sex differences in the adult human brain: evidence from 5216 UK biobank participants. Cereb. Cortex 28, 2959–2975. 10.1093/cercor/bhy109 29771288 PMC6041980

[B104] Robertson C. L. Ishibashi K. Chudzynski J. Mooney L. J. Rawson R. A. Dolezal B. A.. (2016). Effect of exercise training on striatal dopamine D2/D3 receptors in methamphetamine users during behavioral treatment. Neuropsychopharmacology 41, 1629–1636. 10.1038/npp.2015.331 26503310 PMC4832026

[B105] Rönnlund M. Nyberg L. Backman L. Nilsson L. G. (2005). Stability, growth and decline in adult life span development of declarative memory: cross-sectional and longitudinal data from a population-based study. Psychol. Aging 20, 3–18. 10.1037/0882-7974.20.1.3 15769210

[B106] Rosano C. Guralnik J. Pahor M. Glynn N. W. Newman A. B. Ibrahim T. S.. (2017). Hippocampal response to a 24-month physical activity intervention in sedentary older adults. Am. J. Geriatr. Psychiatry 25, 209–217. 10.1016/j.jagp.2016.11.007 27986412 PMC5568026

[B107] Rovio S. Spulber G. Nieminen L. J. Niskanen E. Winblad B. Tuomilehto J.. (2010). The effect of midlife physical activity on structural brain changes in the elderly. Neurobiol. Aging 31, 1927–1936. 10.1016/j.neurobiolaging.2008.10.007 19062136

[B108] Ruscheweyh R. Willemer C. Kruger K. Duning T. Warnecke T. Sommer J.. (2011). Physical activity and memory functions: an interventional study. Neurobiol. Aging 32, 1304–1319. 10.1016/j.neurobiolaging.2009.08.001 19716631

[B109] Salthouse T. A. (2011). Neuroanatomical substrates of age-related cognitive decline. Psychol. Bull. 137, 753–784. 10.1037/a0023262 21463028 PMC3132227

[B110] Scharfman H. E. MacLusky N. J. (2017). Sex differences in hippocampal area CA3 pyramidal cells. J. Neurosci. Res. 95, 563–575. 10.1002/jnr.23927 27870399 PMC5120657

[B111] Sheel A. W. Richards J. C. Foster G. E. Guenette J. A. (2004). Sex differences in respiratory exercise physiology. Sports Med. 34, 567–579. 10.2165/00007256-200434090-00002 15294007

[B112] Shi R. Tian X. Feng Y. Cheng Z. Lu J. Brann D. W.. (2019). Expression of aromatase and synthesis of sex steroid hormones in skeletal muscle following exercise training in ovariectomized rats. Steroids 143, 91–96. 10.1016/j.steroids.2019.01.003 30664864

[B113] Sink K. M. Espeland M. A. Castro C. M. Church T. Cohen R. Dodson J. A.. (2015). Effect of a 24-month physical activity intervention vs health education on cognitive outcomes in sedentary older adults: the LIFE randomized trial. JAMA 314, 781–790. 10.1001/jama.2015.9617 26305648 PMC4698980

[B114] Smith P. J. Blumenthal J. A. Hoffman B. M. Cooper H. Strauman T. A. Welsh-Bohmer K.. (2010). Aerobic exercise and neurocognitive performance: a meta-analytic review of randomized controlled trials. Psychosom. Med. 72, 239–252. 10.1097/psy.0b013e3181d14633 20223924 PMC2897704

[B115] Sofi F. Valecchi D. Bacci D. Abbate R. Gensini G. F. Casini A.. (2011). Physical activity and risk of cognitive decline: a meta-analysis of prospective studies. J. Intern. Med. 269, 107–117. 10.1111/j.1365-2796.2010.02281.x 20831630

[B116] Stroth S. Reinhardt R. K. Thone J. Hille K. Schneider M. Hartel S.. (2010). Impact of aerobic exercise training on cognitive functions and affect associated to the COMT polymorphism in young adults. Neurobiol. Learn. Mem. 94, 364–372. 10.1016/j.nlm.2010.08.003 20800689

[B117] Szuhany K. L. Bugatti M. Otto M. W. (2015). A meta-analytic review of the effects of exercise on brain-derived neurotrophic factor. J. Psychiatr. Res. 60, 56–64. 10.1016/j.jpsychires.2014.10.003 25455510 PMC4314337

[B118] Tamura M. Nemoto K. Kawaguchi A. Kato M. Arai T. Kakuma T.. (2015). Long-term mild-intensity exercise regimen preserves prefrontal cortical volume against aging. Int. J. Geriatr. Psychiatry 30, 686–694. 10.1002/gps.4205 25353992

[B119] ten Brinke L. F. Bolandzadeh N. Nagamatsu L. S. Hsu C. L. Davis J. C. Miran-Khan K.. (2015). Aerobic exercise increases hippocampal volume in older women with probable mild cognitive impairment: a 6-month randomised controlled trial. Br. J. Sports Med. 49, 248–254. 10.1136/bjsports-2013-093184 24711660 PMC4508129

[B120] Terry R. D. Katzman R. (2001). Life span and synapses: will there be a primary senile dementia? Neurobiol. Aging 22, 347–348; discussion 353–354. 10.1016/s0197-4580(00)00250-5 11378236

[B121] Triviño-Paredes J. Patten A. R. Gil-Mohapel J. Christie B. R. (2016). The effects of hormones and physical exercise on hippocampal structural plasticity. Front. Neuroendocrinol. 41, 23–43. 10.1016/j.yfrne.2016.03.001 26989000

[B122] van Uffelen J. G. Chinapaw M. J. Van Mechelen W. Hopman-Rock M. (2008). Walking or vitamin B for cognition in older adults with mild cognitive impairment? A randomised controlled trial. Br. J. Sports Med. 42, 344–351. 10.1136/bjsm.2007.044735 18308888

[B123] Varma V. R. Chuang Y. F. Harris G. C. Tan E. J. Carlson M. C. (2015). Low-intensity daily walking activity is associated with hippocampal volume in older adults. Hippocampus 25, 605–615. 10.1002/hipo.22397 25483019 PMC4425252

[B124] Varma V. R. Tang X. Carlson M. C. (2016). Hippocampal sub-regional shape and physical activity in older adults. Hippocampus 26, 1051–1060. 10.1002/hipo.22586 27009597 PMC6681805

[B125] Vecchio L. M. Meng Y. Xhima K. Lipsman N. Hamani C. Aubert I. (2018). The neuroprotective effects of exercise: maintaining a healthy brain throughout aging. Brain Plast. 4, 17–52. 10.3233/bpl-180069 30564545 PMC6296262

[B126] Voss M. W. Nagamatsu L. S. Liu-Ambrose T. Kramer A. F. (2011). Exercise, brain, and cognition across the life span. J. Appl. Physiol. 111, 1505–1513. 10.1152/japplphysiol.00210.2011 21527670 PMC3220305

[B127] Voss M. W. Prakash R. S. Erickson K. I. Basak C. Chaddock L. Kim J. S.. (2010). Plasticity of brain networks in a randomized intervention trial of exercise training in older adults. Front. Aging Neurosci. 2:32. 10.3389/fnagi.2010.00032 20890449 PMC2947936

[B128] Voss M. W. Vivar C. Kramer A. F. Van Praag H. (2013). Bridging animal and human models of exercise-induced brain plasticity. Trends Cogn. Sci. 17, 525–544. 10.1016/j.tics.2013.08.001 24029446 PMC4565723

[B129] Wagner A. D. Maril A. Bjork R. A. Schacter D. L. (2001). Prefrontal contributions to executive control: fMRI evidence for functional distinctions within lateral prefrontal cortex. Neuroimage 14, 1337–1347. 10.1006/nimg.2001.0936 11707089

[B130] Weinstein A. M. Voss M. W. Prakash R. S. Chaddock L. Szabo A. White S. M.. (2012). The association between aerobic fitness and executive function is mediated by prefrontal cortex volume. Brain Behav. Immun. 26, 811–819. 10.1016/j.bbi.2011.11.008 22172477 PMC3321393

[B131] WHO (2018). Ageing and Health Fact Sheet [Online]. World Health Organization Available online at: http://www.who.int/news-room/fact-sheets/detail/ageing-and-health. Accessed February 5, 2018.

[B132] Young J. Angevaren M. Rusted J. Tabet N. (2015). Aerobic exercise to improve cognitive function in older people without known cognitive impairment. Cochrane Database Syst. Rev. 4:CD005381. 10.1002/14651858.cd005381.pub4 25900537 PMC10554155

[B133] Yüksel O. Ateş M. Kızıldağ S. Yüce Z. Koç B. Kandiş S.. (2019). Regular aerobic voluntary exercise increased oxytocin in female mice: the cause of decreased anxiety and increased empathy-like behaviors. Balkan Med. J. 36, 257–262. 10.4274/balkanmedj.galenos.2019.2018.12.87 31140236 PMC6711252

